# Intermittent Fasting: Benefits, Side Effects, Quality of Life, and Knowledge of the Saudi Population

**DOI:** 10.7759/cureus.34722

**Published:** 2023-02-07

**Authors:** Hani Shalabi, Abdulrahman S Hassan, Faris A AL-Zahrani, Abdullah H Alarbeidi, Mohammed Mesawa, Hisham Rizk, Abrar A Aljubayri

**Affiliations:** 1 Internal Medicine, University of Jeddah, Jeddah, SAU; 2 Medicine and Surgery, University of Jeddah, Jeddah, SAU; 3 General Surgery, Faculty of Medicine, University of Jeddah, Jeddah, SAU

**Keywords:** quality of life, obesity, diet, weight loss, intermittent fasting

## Abstract

Introduction

Intermittent fasting (IF) is an eating pattern that alternates between periods of fasting and eating. IF has shown many benefits for people who are obese and are trying to lose weight and attain a healthy lifestyle. The aim of our study was to evaluate the efficacy of IF and how it can be used as a daily lifestyle as well as to measure the knowledge of the IF diet among the Saudi population about its benefits, side effects, and life quality.

Method

For this retrospective cross-sectional study, data about the common side effects, benefits, and the measurement of the quality of life were collected by a survey distributed using Google Forms. Microsoft Excel was used for the data analysis, with the data and results being mainly expressed as numbers and percentages.

Results

Among the 147 individuals practicing the IF plan who were surveyed, male participants were more than females (53.7% vs 46.3%). The highest percentage of respondents were in the age group 18-35 years old, and 88 individuals (59.9%) had a high body mass index (BMI). Duration of fasting varied from less than a month to three months in 70.8%, and 71.4% of participants had undertaken IF several times. Side effects were headache (61.3%), lethargy (68%), mood swings (57.8%), and lastly dizziness and polyuria (55.8% and 46.2%, respectively). Slightly more females expressed happiness than males (86.8% vs 83.6%).

Conclusion

The IF diet is an efficient dietary plan for those aiming at a weight loss journey over a short duration, ranging from less than a month up to three months. Minimal side effects were found during fasting, being of different intensities, which did not need surgical or medical treatment. All in all, most of our respondents were pleased with their experience and saw excellent weight loss results using the IF diet.

## Introduction

Fasting is a practice followed by our ancestors for thousands of years; it has several aspects and is of different types such as for religious, cultural, or health reasons. Muslims fast during the month of Ramadan for a specific period during the day, which is similar to intermittent fasting (IF) [1]. This type of diet is based on an eating pattern that alternates between periods of fasting and eating. IF on the other hand mainly focuses on the timing of the meal, yet the quality of the food is also considered a factor, since losing weight is the primary goal. It commonly consists of a daily fast for 16 hours, a 24-hour fast on alternate days, or a fast two days per week on non-consecutive days. These types of diets aren't only used for obese people trying to lose weight, but also for people with cardiovascular risk factors and type 2 diabetes risk factors [2].

Obesity is a major medical problem that can lead to other significant risks such as metabolic diseases, mainly type 2 diabetes, hypertension, and stroke, and gastrointestinal tract diseases such as the fatty liver. According to WHO, obesity has nearly tripled between 1975 and 2016, with 39% of adults aged 18 years and over being overweight in 2016, and 13% obese [3]. Preventable obesity is still considered a major problem around the world.

IF provides many benefits for the obese and those trying to lose weight and attain a healthy lifestyle. It can reduce body fat and inflammation, and improve glucose metabolism [4]. Indeed, although IF is used for weight loss, ADF (alternate day fasting), which is a type of IF, has shown benefits in non-obese people also by lowering triacylglycerol, C reactive protein, and leptin, while increasing low-density lipoprotein particle size and adiponectin concentrations [5].

As much as IF is filled with benefits, there are mild side effects that can happen during fasting, which do not generally require medical or surgical treatment. Possible side effects can include dizziness, nausea, insomnia, headache, weakness, etc. [6]. Another study has shown low blood sugar (hypoglycemia) to be a side effect of IF [7].

The aim of our cross-sectional study was to observe the effectiveness of IF throughout the Saudi population. Furthermore, we wanted to evaluate behavioral characteristics and experiences of the IF diet from people's viewpoints on the side effects, benefits, and life quality.

## Materials and methods

Study design and period

A retrospective cross-sectional study was carried out among individuals who had or are currently following the IF. In the four-week preparation period, we created our study title, reviewed the literature to gather information on typical side effects and how to measure the quality of life, and created a questionnaire. In the following four weeks, we completed our research, which included looking for the target group on social media, creating a Google Form for the survey, and testing the form by using a small sample of the findings to make sure they presented as we planned. We collected and examined our data for a further four weeks before writing our report.

Inclusion and exclusion criteria

The study includes people who have ever practiced or are currently practicing IF and who can speak the Arabic language. The target population is in Saudi Arabia in all regions of the kingdom. The initial assessment questions excluded participants who had never engaged in IF. The sample size was calculated automatically using Google Forms. A total of 300 people were registered in the Google Form; 147 of them had practiced IF, and 153 were excluded.

Data collection tool

We developed a questionnaire in Arabic to collect data about IF attitudes and quality of life (questionnaire in the Appendices section). The questionnaire was developed to meet the health-related quality of life (HRQOL) measures, which were translated into Arabic to be suitable for the participant's understanding of the question in our survey. The Centers for Disease Control and Prevention (CDC) HRQOL-4 measures had acceptable test-retest reliability and strong internal validity, which has been used by the CDC and its partners, for tracking population health status and HRQOL measures in states and communities [8]. The standard four-item set of healthy days’ core questions was developed by the CDC. The questionnaire consists of the following four main parts: (i) sociodemographic data, (ii) side effects assessment, (iii) participants' attitude toward IF assessment, and (iv) participants' quality of life assessment.

Data collection technique

We conducted the survey in an electronic self-assessment format using Google Forms. We targeted IF groups on social media in Saudi Arabia for a time interval of four weeks through messages and direct contact with the group administrator and ensured that only people who practiced IF participated in the survey. One of the first questions in the survey was “Have you tried Intermittent fasting?” If the answer was no, then the participant is excluded from the survey. The data obtained from the survey were reviewed and automatically copied into a personal computer.

Data entry and analysis

The data collected using the questionnaire was rearranged in Microsoft Excel data sheets. The data was mainly expressed as numbers and percentages. We used a chi-square test to evaluate participants’ perceived happiness, which also indicated the p-value for statistical significance. We also used a pie chart that displays the change in the participants’ body weight following their adoption of IF and to seek the perceived happiness of the participants' IF experience.

Ethical considerations

This study was approved by the Bioethics Committee for Scientific and Medical Research at the University of Jeddah (Approval number UJ-REC-069). Individual consent was required prior to data collection, and it was stated on the questionnaire's front page that completing it signified consent to participate in the study. All information was kept private and was solely utilized for scientific studies. Furthermore, we made sure that parental consent was given to participants less than 18 years old.

## Results

A total of 147 respondents who practiced the IF diet plan participated to explore their IF experience and to determine its consequences on their overall health.

Table 1 shows the respondent characteristics of gender, age, and body mass index (BMI). Male participants were more than the females (53.7% vs 46.3%), and 75.5% were in the age group of 18-35 years. According to the BMI ranges they were derived from CDC, 38.1% of the participants were within the normal weight category, with 35.4% being overweight, and 23.1% obese.

**Table 1:** The characteristics of the respondents (n=147) BMI: body mass index CDC: Centers for Disease Control and Prevention The BMI ranges were taken from the Centers for Disease Control and Prevention [9].

Characteristics		Number	Percentage
Gender	Male	79	53.70%
	Female	68	46.30%
Age categories	<18 years	4	2.40%
	18-35 years	111	75.50%
	36-55 years	27	18.40%
	56-70 years	5	3.40%
	> 70 years	N/A	N/A
BMI categories	Within Normal (18.5-24.9)	56	38.1%
	Overweight (25-29.9)	52	35.40%
	Obesity (30-39.9)	34	23.1%
	Morbid Obesity (≥40)	2	1.40%
	underweight (<18.5)	3	2.00%

As seen in Table 2, most of the respondent individuals (70.8%) practiced the IF diet for a duration ranging from less than a month to three months, while 29.3% of the individuals practiced the diet from more than three months to six months. According to the frequency, 28.6% were for the first time experiencing IF, and for the second time was 29.2%. Lastly, 34% have experienced IF more than three times. During the IF diet plan, close to two-thirds of the respondents (67.3%) were performing physical exercise and 32.7% didn't perform any physical exercise.

**Table 2:** The practice of intermittent fasting (n=147)

Practices during Intermittent fasting		Number	Percentage
Duration	<1 month	47	32%
	1 month	36	24.50%
	> 1 month-3 months	21	14.30%
	>3 month-6 months	43	29.20%
Frequency	Once	42	28.60%
	Twice	43	29.20%
	3 times	12	8.20%
	>3 times	50	34%
Regular Performance of Physical Exercise	Yes	99	67.30%
	No	48	32.70%

**Figure 1:**
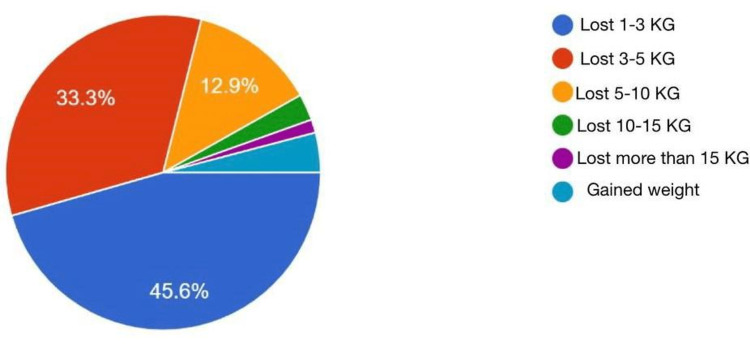
Change in weight as a response to intermittent fasting

Figure 1* *displays the weight changes in a sample who practiced IF throughout their lifetime so far, of whom 67 respondents (45.6%) reported weight loss varying from 1 kg to less than 3 kg. Of these, 49 respondents (33.3%) lost 3-5 kg and 19 (12.9%) respondents lost 5-10 kg; four respondents (2.7%) reported losing 10-15 kg, whereas only two respondents (1.4%) reported losing more than 15 kg. Lastly, six respondents (4.1%) reported weight gain.

We asked responders about any symptoms they may have felt while following the IF diet allowed researchers to gauge the overall effectiveness of the diet, particularly within the first month (Table 3). As shown, the most frequently occurring symptoms were headache (mild 36.1%, moderate 17.7%, and severe 7.5%), lethargy (mild 27.9%, moderate 23.1%, and severe 17%), mood swings (mild 31.3%, moderate 16.3%, and severe 10.2%), and dizziness with a total of 55.8, and lastly, polyuria among 46.2% respondents.

**Table 3:** Symptoms within the first month of adopting the intermittent fasting diet

Symptoms	Mild	Moderate	Severe	Not Sure	Not at All
Headache	53 (36.1%)	26 (17.7%)	11 (7.5%)	17 (11.6%)	40 (27.2%)
Mood swings	46 (31.3%)	24 (16.3%)	15 (10.2%)	19 (12.9%)	43 (29.3%)
Palpitations	24 (16.3%)	18 (12.2%)	11 (7.5%)	16 (10.9%)	78 (53.1%)
Fever	10 (6.8%)	4 (2.7%)	4 (2.7%)	10 (6.8%)	119 (81%)
Flu	18 (12.2%)	3 (2%)	7 (4.8%)	11 (7.5%)	108 (73.5%)
Low Blood Sugar	18 (12.2%)	15 (10.2%)	9 (6.1%)	26 (17.7%)	79 (53.7%)
Lethargy	41 (27.9%)	34 (23.1%)	25 (17%)	4 (2.7%)	43 (29.3%)
Constipation	29 (19.7%)	19 (12.9%)	9 (6.1%)	15 (10.2%)	75 (51%)
Dizziness	41 (27.9%)	25 (17%)	16 (10.9%)	9 (6.1%)	56 (38.1%)
Vomiting	16 (10.9%)	6 (4.1%)	4 (2.7%)	8 (5.4%)	113 (76.9%)
Dehydration	27 (18.4%)	18 (12.2%)	5 (3.4%)	8 (5.4%)	89 (60.5%)
Bloating	17 (11.6%)	10 (6.8%)	9 (6.1%)	15 (10.2%)	96 (65.3%)
Polyuria	29 (19.7%)	25 (17%)	14 (9.5%)	15 (10.2%)	64 (43.5%)

**Figure 2:**
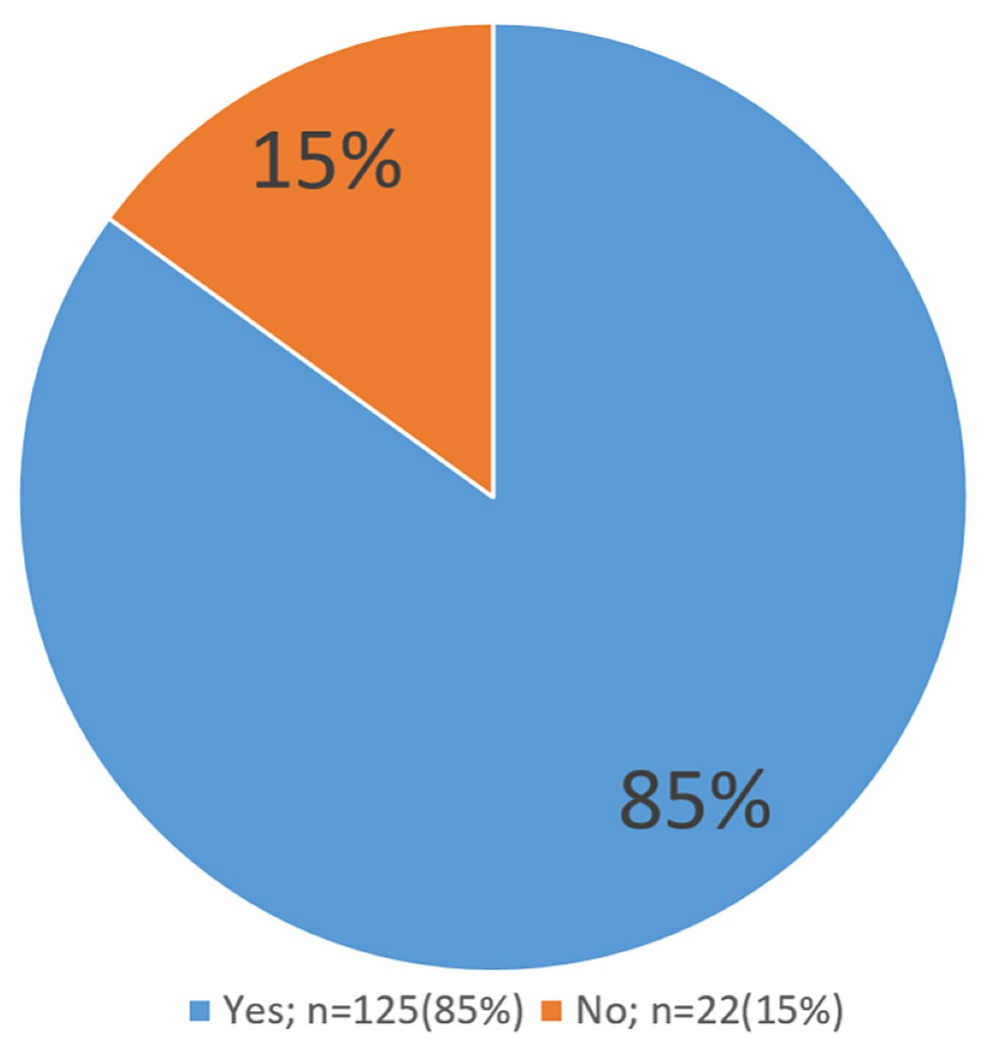
Perceived happiness regarding intermittent fasting

Figure 2 demonstrates a pie-chart of perceived happiness for the IF experience of our respondents, with 85% of them pleased about adopting the IF diet and 15% of them weren't.

According to gender in Table 4, females were happier than males (86.8% vs 83.6%), and when it came to the age groups we found that the most perceived happiness was in the age group 36-55 years old (92.6%). In the BMI category happiness perception among obese respondents had the highest percentage (88.2%). These differences showed no statistical significance (p > 0.05).

**Table 4:** Perceived happiness about the intermittent fasting diet according to the characteristics (gender, BMI categories, and age) of the respondents * Based on the chi-square test BMI: body mass index

Variables		Yes		No		X^2	P-Value
		Number	%	Number	%		
Gender	Male	66	83.6%	13	16.4%	0.297	0.585
	Female	59	86.8%	9	13.2%		
Age	<18 Years	3	75%	1	25%	2.074	0.149
	18-35 Years	91	82%	20	18		
	36-55 Years	25	92.6%	2	7.4%		
	56-70 Years	4	80%	1	20%		
	> 70 Years	N/A	N/A	N/A	N/A		
BMI categories	Within Normal	49	87.5%	7	12.5%	3.489	0.061
	Overweight	43	82.7%	9	17.3%		
	Obesity	30	88.2%	4	11.7%		
	Morbid Obesity	1	50%	1	50%		
	Underweight	2	66.6%	1	33.3%		

## Discussion

IF has shown an increase in popularity as a method for weight loss and health optimization, and previous research has found promising results for its utility [10]. The current study collected data on IF to perceive its efficacy, side effects, and benefits, as well as the measurement of the resulting quality of life among the Saudi population adopting IF.

The findings of our study showed that the number of males was slightly higher than the females by 7.4%, while another study has shown that female participants tend to be a majority, and also practice IF longer than males [1]. When it comes to age category, most of our respondents were between the age of 18 and 35, which makes sense since this age group is adolescents/adults who have greater concern about their weight than any other age category. The relationship between weight loss and young adults was studied by Jessica Gokee LaRose et al., who stated that people <35 years old have a higher chance of gaining weight than older adults [11].

Most of our respondents had a high BMI; a combination of overweight (35.4%), obese (23.1%), and morbidly obese (1.4%). Among the respondents, 59.9% had a high BMI and 38.1% were within the normal weight, with the remaining 2% underweight. So, most of these participants practiced IF to lose weight and reduce their BMI number. A study by Maha H Alhussain et al. in Saudi Arabia compared the usage of IF for different purposes and found that almost half of the respondents (47.59%) were trying to lose weight [12].

When it came to the duration, people who practiced the IF diet for less than a month and up to three months constituted 70.8%, while those who did it for a long term (>3-6 months) were 29.3%. Frequency-wise, the percentages were relatively similar to one another; the people who practiced it once were 28.6%, twice 29.3%, and thrice were only 8.2% making it the least, whereas those adopting IF more than three times were 34%, accounting for the largest percentage.

Moreover, our statistics show that 67.3% of the people who adopted the IF diet also performed physical exercise, whereas only 32.7% practiced IF without any physical exercise. People felt that exercising while fasting isn’t a great option since you aren’t eating, yet a study has shown that exercising while you are on your fast increases adipose tissue lipolysis and peripheral fat oxidation, both of which act as energy producers for the body [13]. Alexandra Ferreira Vieira found that those who did moderate aerobic workouts during the fasting state showed a significant increase in fat oxidation [14].

A total of 94.5% of our respondents have lost weight (ranging from 1 kg up to 10 kg), and 4.1% had a significant decrease in weight (greater than or equal to 10 kg). Only six of our participants gained weight during their IF, which might be due to the quality of the food they chose to eat. Lora E. Burke et al. carried out a systematic review of 22 studies with self-monitoring; 15 out of the 22 studies involved dietary self-monitoring. Within these 22 studies, they found out there was more weight loss among people who self-monitored than among the less frequent self-monitoring group [15].

In spite of the fact that IF may be used by many people to cleanse their bodies or for weight loss, yet the diet does come with side effects, which you may experience within the first month of your fast. The most frequent side effects were headache, dizziness, polyuria, mood swings, and lastly lethargy. All of these symptoms had different intensity levels, from mild to severe. Headache is a common side effect seen in fasting in general, which is mainly due to hypoglycemia, and it is characterized as a diffuse and non-pulsatile headache. The headache is of mild to moderate intensity, it happens during the fasting period, and when you fast at least for 8 hours [16].

Lastly, 85% of respondents were happy with their results, and only 15% weren’t happy with their IF experience. As mentioned before, this may be due to their nutritional lifestyle routine or because they did not achieve the desired results.

The study has some limitations. First, it was distributed over social media groups with a special interest in the IF diet, thereby possibly exaggerating the positive attitude toward this diet. Second, the study's retrospective questions may have been influenced by how participants overall felt and how they remembered their symptoms.

## Conclusions

The majority of our participants tried the IF diet ranging from a month up to three months, suggesting that this type of diet is a short-term solution for people trying to lose weight. Almost all of the participants lost weight. Side effects were reported in our results, with a variation of intensities (mild, moderate, severe), particularly within the first month. All in all, the majority of our respondents were pleased with their experience and saw excellent weight loss results with the IF diet.

## Appendices

Intermittent fasting questionnaire ENGLISH

We, a group of medical students, inviting you to participate in a questionnaire to measure the community's awareness of intermittent fasting.

This questionnaire was designed by researchers at Jeddah University/College of Medicine

.sa under the supervision of Dr. Hisham Rizk

All information will be saved with the principal investigator. Access to the data will be restricted to the research team and responses to questions will be kept confidential. It will be used for research purposes only and will not be shared with anyone. Participants under 18 parental consent are needed.

- Please note that once you have answered the questionnaire, it means that you have understood the above and have given your consent to share your data with the researcher.*

o Agree

o Disagree

- Have you tried Intermittent fasting?*

o Yes

o No

Section 1 - Tried Intermittent fasting

- Age*

o Under 18 years

o 18-35 years

o 36-55 years

o 56-70 years

o Over 70 years

- Sex*

o Male

o Female

- Height (centimeter): …………………

- Weight (kg): …………………

1 - How long have you been practicing Intermittent fasting?*

o Less than a month

o 1 month

o 1-3 month

o 3-6 month

2 - How many times have you followed Intermittent fasting?*

o Once

o Twice

o Three times

o Many times

3 - Did you exercise while you were following Intermittent fasting?*

o No, I did not exercise

o Yes, I exercised

4 - How much weight did you lose during the first month?*

o My weight has not changed

o Lost 1-3 kg

o Lost 3-5 kg

o Lost 5-10 kg

o Lost 10-15 kg

o Lost >15 kg

o I gained weight

5 - Within the first month of Intermittent fasting, did you suffer from headaches?*

o I did not at all

o Mild

o Moderate

o Severe

o Not sure

6 - Within the first month of Intermittent fasting, did you notice any mood swings?*

o I did not at all

o Mild

o Moderate

o Severe

o Not sure

7 - Within the first month of Intermittent fasting, did you notice an increase in heart rate (palpitations)?*

o I did not at all

o Mild

o Moderate

o Severe

o Not sure

8 - Within the first month of Intermittent fasting, did you experience any fever?*

o I did not at all

o Mild

o Moderate

o Severe

o Not sure

9 - Within the first month of Intermittent fasting, did you suffer from cold or flu symptoms?*

o I did not at all

o Mild

o Moderate

o Severe

o Not sure

10 - Within the first month of Intermittent fasting, did you experience a drop in blood sugar?*

o I did not at all

o Mild

o Moderate

o Severe

o Not sure

11 - Within the first month of Intermittent fasting, did you suffer from lethargy?*

o I did not at all

o Mild

o Moderate

o Severe

o Not sure

12 - Within the first month of Intermittent fasting, did you suffer from constipation?*

o I did not at all

o Mild

o Moderate

o Severe

o Not sure

13 - Within the first month of your Intermittent fasting, did you experience dizziness or lightheadedness?*

o I did not at all

o Mild

o Moderate

o Severe

o Not sure

14 Within the first month of Intermittent fasting, did you suffer from vomiting?*

o I did not at all

o Mild

o Moderate

o Severe

o Not sure

15 - Within the first month of Intermittent fasting, did you suffer from Dehydration?*

o I did not at all

o Mild

o Moderate

o Severe

o Not sure

16 - Within the first month of Intermittent fasting, did you suffer from bloating?

o I did not at all

o Mild

o Moderate

o Severe

o Not sure

17 - Within the first month of Intermittent fasting, did you suffer from Polyuria?*

o I did not at all

o Mild

o Moderate

o Severe

o Not sure

18 - When did the symptoms resolve?*

o First 24 hours

o First 3 days

o First week

o Not sure

19 - Have you had any other symptoms? Please mention it:

……………………………………………………………………………………………………………………………………………………………………………………………………………………………………………………

20 - Are you happy with your current health on Intermittent fasting?*

o Yes

o No

21 - Would you recommend Intermittent fasting to someone who wants to lose weight?*

o Yes

o No

o Not Sure

Section 2 - No Intermittent fasting

 - Age*

o Under 18 years

o 35-18 years

o 36-55 years

o 56-70 years

o Over 70 years

- Sex*

o Male

o Female

Height (centimeter): …………………

Weight (kg): …………………

## References

[1] Alnasser A, Almutairi M (2022). Considering intermittent fasting among Saudis: insights into practices. BMC Public Health.

[2] Welton S, Minty R, O'Driscoll T, Willms H, Poirier D, Madden S, Kelly L (2020). Intermittent fasting and weight loss: systematic review. Can Fam Physician.

[3] (2022). World Health Organization, "Health Topics". https://www.who.int/news-room/fact-sheets/detail/obesity-and-overweight.

[4] Malinowski B, Zalewska K, Węsierska A (2019). Intermittent fasting in cardiovascular disorders - an overview. Nutrients.

[5] Varady KA, Bhutani S, Klempel MC (2013). Alternate day fasting for weight loss in normal weight and overweight subjects: a randomized controlled trial. Nutr J.

[6] Grajower MM, Horne BD (2019). Clinical management of intermittent fasting in patients with diabetes mellitus. Nutrients.

[7] Vasim I, Majeed CN, DeBoer MD (2022). Intermittent fasting and metabolic health. Nutrients.

[8] (2023). Measuring Healthy Days: Population Assessment of Health-related Quality of Life. https://stacks.cdc.gov/view/cdc/6406.

[9] (2023). Defining Adult Overweight & Obesity. https://www.cdc.gov/obesity/basics/adult-defining.html.

[10] Wilhelmi de Toledo F, Grundler F, Sirtori CR, Ruscica M (2020). Unravelling the health effects of fasting: a long road from obesity treatment to healthy life span increase and improved cognition. Ann Med.

[11] LaRose JG, Leahey TM, Hill JO, Wing RR (2013). Differences in motivations and weight loss behaviors in young adults and older adults in the National Weight Control Registry. Obesity (Silver Spring).

[12] Alhussain MH, Almarri DM, Arzoo S (2021). Study on the effect of intermittent fasting on body mass index, physical activity and sleep in adults. J Clin Diagn Res.

[13] Zouhal H, Saeidi A, Salhi A (2020). Exercise training and fasting: current insights. Open Access J Sports Med.

[14] Vieira AF, Costa RR, Macedo RC, Coconcelli L, Kruel LF (2016). Effects of aerobic exercise performed in fasted v. fed state on fat and carbohydrate metabolism in adults: a systematic review and meta-analysis. Br J Nutr.

[15] Burke LE, Wang J, Sevick MA (2011). Self-monitoring in weight loss: a systematic review of the literature. J Am Diet Assoc.

[16] Soares AA, de Vasconcelos CAC, Silva-Néto RP (2018). Headaches and food abstinence: a review. J Clin Case Stu.

